# Immunological role and prognostic value of somatostatin receptor family members in colon adenocarcinoma

**DOI:** 10.3389/fphar.2023.1255809

**Published:** 2023-10-13

**Authors:** Xiaoqian Yu, Xuejie Yang, Hui Nie, Wenying Jiang, Xiaoyun He, Chunlin Ou

**Affiliations:** ^1^ Department of Pathology, Xiangya Hospital, Central South University, Changsha, Hunan, China; ^2^ Department of Oncology, Xiangya Hospital, Central South University, Changsha, Hunan, China; ^3^ Departments of Ultrasound Imaging, Xiangya Hospital, Central South University, Changsha, Hunan, China; ^4^ National Clinical Research Center for Geriatric Disorders, Xiangya Hospital, Central South University, Changsha, China

**Keywords:** SSTR family, colon adenocarcinoma, prognosis, immune infiltration, immunotherapy, therapeutic target

## Abstract

Colon adenocarcinoma (COAD) is among the most prevalent cancers worldwide, ranking as the third most prevalent malignancy in incidence and mortality. The somatostatin receptor (SSTR) family comprises G-protein-coupled receptors (GPCRs), which couple to inhibitory G proteins (Gi and Go) upon binding to somatostatin (SST) analogs. GPCRs are involved in hormone release, neurotransmission, cell growth inhibition, and cancer suppression. However, their roles in COAD remain unclear. This study used bioinformatics to investigate the expression, prognosis, gene alterations, functional enrichment, and immunoregulatory effects of the SSTR family members in COAD. SSTR1-4 are differentially downregulated in COAD, and low SSTR2 expression indicates poor survival. Biological processes and gene expression enrichment of the SSTR family in COAD were further analyzed using the Kyoto Encyclopedia of Genes and Genomes and Gene Ontology. A strong correlation was observed between SSTR expression and immune cell infiltration. We also quantified SSTR2 expression in 25 COAD samples and adjacent normal tissues using quantitative real-time polymerase chain reaction. We analyzed its correlation with the dendritic cell–integrin subunit alpha X marker gene. The biomarker exploration of the solid tumors portal was used to confirm the correlation between SSTR2 with immunomodulators and immunotherapy responses. Our results identify SSTR2 as a promising target for COAD immunotherapy. Our findings provide new insights into the biological functions of the SSTR family and their implications for the prognosis of COAD.

## Introduction

Colorectal cancer (CRC) is a common malignancy worldwide, ranking third among all malignancies in incidence and mortality (Shaukat and Levin, 2022). Colonic adenocarcinoma (COAD) is the most common form of CRC (Mutch, 2007). COAD progresses through several stages, from normal mucosa to adenoma, and finally to cancer (Riihimäki et al., 2016; Arnold et al., 2017). The usual presentation of COAD during medical treatment includes changes in bowel movements; in addition to the onset of rectal bleeding, iron-deficiency anemia, abdominal pain, weight loss, and loss of appetite (Thanikachalam and Khan, 2019); there are no typical or specific clinical signs. However, comprehensive testing is lacking in most areas. Consequently, patients with COAD are commonly diagnosed with advanced cancer, making treatment more challenging and affecting their prognosis (Raza et al., 2022). Therefore, the identification of new biomarkers and therapeutic targets to improve the survival of patients with COAD is urgently needed.

Somatostatin (SST) is an inhibitory peptide hormone produced by neuroendocrine, inflammatory, and immune cells found in the central nervous system (CNS) and in several peripheral tissues in response to cytokines, growth factors, thyroid and steroid hormones, neurotransmitters, neuropeptides, nutrients, and ions (Patel, 1999; Wu et al., 2020). SST binds to certain cell surface receptors to suppress exocrine and endocrine secretions as well as tumor cell growth (Priyadarshini et al., 2022). Five different subtypes of SST receptors (SSTRs) (SSTR1-5) have been identified. SSTR is a member of the family of G protein-coupled receptors. The five isoforms are coupled with the Gi protein, which can affect the concentration of intracellular cyclic AMP (cAMP) by regulating the activity of adenylate cyclase and transmitting exogenous signals to cells (Bo et al., 2022; Liguz-Lecznar et al., 2022). SSTRs, which help release hormones, transmit nerve signals, arrest cell growth, and inhibit cancer, are found in abundance in the CNS and associated malignant cells as well as within the peripheral organs, pancreas, and gut (Rorsman and Huising, 2018; Harda et al., 2020). Neuroendocrine tumors (NETs) are a diverse category of tumors that can arise in the digestive tract, lungs, and pituitary among other organs (Klöppel, 2017). The expressions of SSTRs, which act as therapeutic targets for SST analogs, which can slow tumor growth and suppress hormone overproduction, are one inherent characteristic of NETs. Moreover, the degree of SSTR expression in several NETs has predictive significance for treatment response (Rogoza et al., 2022). Moreover, researchers found that the internalization capacity of SSTR and the development of radiolabeled somatostatin analogs have improved cancer diagnosis and treatment (Fani et al., 2017; Delpassand et al., 2022).

However, limited research has examined the expression, prognosis, and immune characteristics of the SSTR family members in COAD. Using research databases and bioinformatic methods, we examined SSTR family gene expressions and their link to clinical features in COAD. Our findings offer new knowledge about the prognosis and biological roles of SSTRs in COAD.

## Materials and methods

### Tumor immune estimation resource database

The Tumor Immune Estimation Resource version 2.0 (TIMER2.0) database (https://cistrome.shinyapps.io/timer/) has three main components: vaccination, exploration, and assessment. TIMER2.0 can analyze relationships between genes and infiltrating cells, compare genetic expression in tumors and normal tissues across different malignant tumor types, and provide easy-to-use interactive visualizations to help explore the data (Li et al., 2020). Using the TIMER2.0 database, we analyzed SSTR family expression in 41 normal and 457 COAD samples. Additionally, TIMER2.0 was employed to examine associations between the mRNA expression of SSTR family members and cells of the COAD immune infiltrate, including CD4^+^ and CD8^+^ T cells, neutrophils, macrophages, dendritic cells (DCs), and B cells. The R-value of the correlation was determined by Spearman’s algorithm with adjustment for tumor purity. Values of *p* < 0.05 were considered significant.

### Human protein atlas

Protein expression information for different cancer types based on immunohistochemical (IHC) analysis is available on the Human Protein Atlas (HPA) portal (http://www.proteinatlas.org). It is a critical resource for many biomedical research projects (Pontén et al., 2011). In this investigation, we conducted IHC to analyze the SSTR family members’ protein expressions in normal and COAD tissues.

### University of Alabama at birmingham cancer data analysis portal

University of Alabama at Birmingham Cancer Data Analysis Portal (UALCAN) (https://ualcan.path.uab.edu) makes it simple to perform Kaplan-Meier survival analyses depending on tumor subgroups, promoter DNA methylation status, and pre-calculated gene/protein expression (Chandrashekar et al., 2017). A stratified analysis was performed based on the individual patient’s cancer stage and nodal status. Student’s t-test was used, and values of *p* < 0.05 were considered to be statistically significant.

### CBioPortal

As a tool for interactively exploring multi-dimensional cancer genomic data sets, cBio Cancer Genomics Portal (http://cbioportal.org) is freely available (Cerami et al., 2012). We used cBioPortal to retrieve a dataset of 594 patients with COAD and conducted co-expression and gene alteration analyses of the SSTR family members.

### STRING database

Known and predicted protein–protein association data of a large number of species are present in the STRING database (https://cn.string-db.org/). The database includes the physical interactions, functional links, and confidence levels that indicate their reliability. The STRING database was used to evaluate the SSTR gene correlations.

### Cytoscape database

High-throughput expression data, other molecular states, and biomolecule interaction networks may be combined using the Cytoscape (http://cbioportal.org) open-source software project (Otasek et al., 2019). A total of 178 commonly mutated SSTR family genes were functionally integrated using Cytoscape after being screened from the cBioPortal database. Node size represents the degree values between these interacting proteins. Larger circles indicate a higher degree of interaction.

### Metascape database

By leveraging more than 40 independent knowledge sources in one integrated platform, Metascape (https://metascape.org) integrates membership search, gene annotation, interactome analysis, and functional enrichment factors (Zhou et al., 2019). The SSTR family–related Gene Ontology (GO) and Kyoto Encyclopedia of Genes and Genomes (KEGG) pathway enrichment analysis was conducted using Metascape.

### PrognoScan

The correlation between SSTR expression and survival in COAD was analyzed using the PrognoScan database (http://www.abren.net/PrognoScan/) (Mizuno et al., 2009). PrognoScan offers a huge collection of freely accessible, clinically annotated cancer microarray datasets that may be applied to assess the biological links between gene expression and patient prognosis. Values of *p* < 0.05 were considered statistically significant.

### Biomarker exploration of solid tumors

Validation was conducted using the Biomarker Exploration of Solid Tumors (BEST) portal (https://rookieutopia.com/app_direct/BEST/). BEST was used to analyze the association between the SSTR family and immunotherapy response and prognosis in COAD.

### Tissue samples

Twenty-five pairs of matched adjacent normal tissue samples and paraffin-embedded archival colon cancer specimens were obtained from Xiangya Hospital (Changsha, P. R. China). None of the patients had received any form of therapy, such as chemotherapy, radiotherapy, or immunotherapy, before the resection. The Xiangya Hospital of Central South University’s research ethics committee approved the collection of clinical colon cancer specimens.

### Isolation of RNA from formalin-fixed and paraffin-embedded samples

After the deparaffinization of formalin-fixed paraffin-embedded (FFPE) colon cancer or normal samples with xylene, total RNA was extracted using total RNA AmoyDx^®^ FFPE RNA Extraction Kit (Cat. #8.02.0019; AmoyDx, Xiamen, P. R. China).

### Quantitative real-time polymerase chain reaction

As discussed previously, quantitative real-time polymerase chain reaction (qRT-PCR) was performed following the RNA extraction and amplification (Ou et al., 2020; He et al., 2021). Table 1 displays the qRT-PCR primer sequences.

**TABLE 1:** Primer sequence for qRT-PCR.

Gene	Primer (forward)	Primer (reverse)
SSTR2	TGG​CTA​TCC​ATT​CCA​TTT​GAC​C	AGG​ACT​GCA​TTG​CTT​GTC​AGG
ITGAX	GGG​ATG​CCG​CCA​AAA​TTC​TC	ATT​GCA​TAG​CGG​ATG​ATG​CCT
U6	CTCGCTTCGGCAGCACA	AAC​GCT​TCA​CGA​ATT​TGC​GT

### Statistical analysis

The statistics for the survival analysis were obtained using the log-rank test, and the associations of the SSTR family with immune infiltration and markers of immune cell type were evaluated using Spearman’s correlation. Student’s t-test was conducted to contrast data from two independent samples. Values of *p* < 0.05 were considered statistically significant.

## Results

### Aberrant expression of SSTR family members in COAD

**FIGURE 1:**
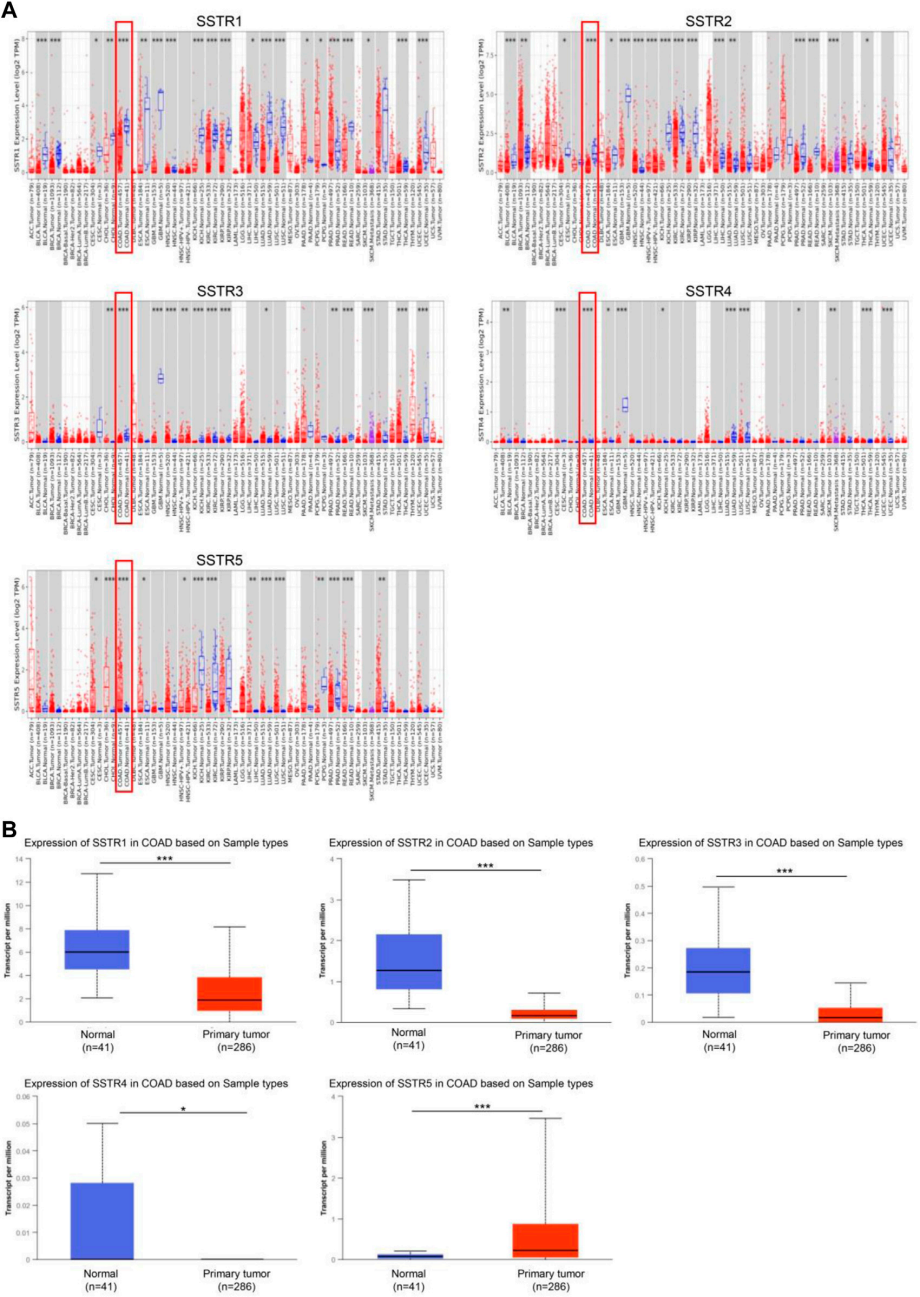
SSTR family members expressed in COAD. **(A)** SSTR expressions in pan-cancer. **(B)** SSTR expressions in COAD. *p < 0.05, **p < 0.01, ***p < 0.001 compared with control. COAD, colon adenocarcinoma; SSTRs, somatostatin receptors.

To investigate the changes in SSTR expression levels in various malignant tissues compared to normal tissues, we employed the TIMER2.0 database to assess the SSTR transcript levels. In COAD tissues, SSTR1-4 mRNA expression levels were remarkably downregulated. However, SSTR5 expression was upregulated (Figure 1A). Easy access to pre-calculated gene and protein expression based on tumor subgroups was provided by UALCAN (https://ualcan.path.uab.edu). This was applied to investigate the SSTR family gene mRNA expression. Our findings revealed that all SSTRs except SSTR5 were significantly downregulated in COAD versus normal controls (*p* < 0.05) (Figure 1B).

**FIGURE 2:**
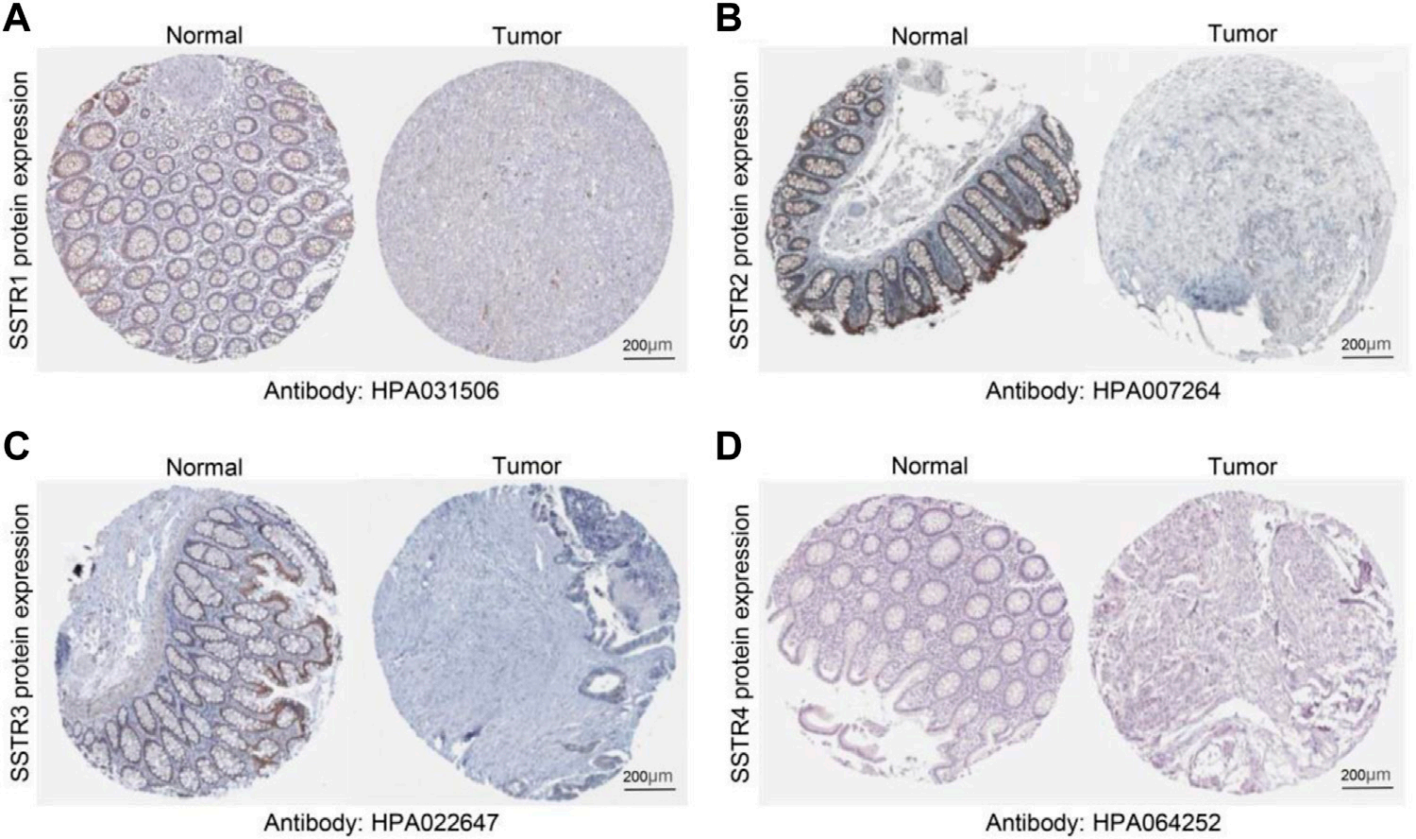
Protein expression levels of SSTR family members in COAD. **(A–D)** Protein expression levels of SSTR1-4 in COAD versus non-cancerous tissues. COAD, colon adenocarcinoma; SSTR, somatostatin receptor.

To verify these results, we examined the SSTR family immunohistochemistry (IHC) results from the Human Protein Atlas database. SSTR1-4 protein levels were lower in COAD versus normal tissues (Figures 2A–D). These outcomes agreed with our earlier mRNA expression research findings.

### Correlation of SSTR family expression with clinicopathological features of COAD

**FIGURE 3:**
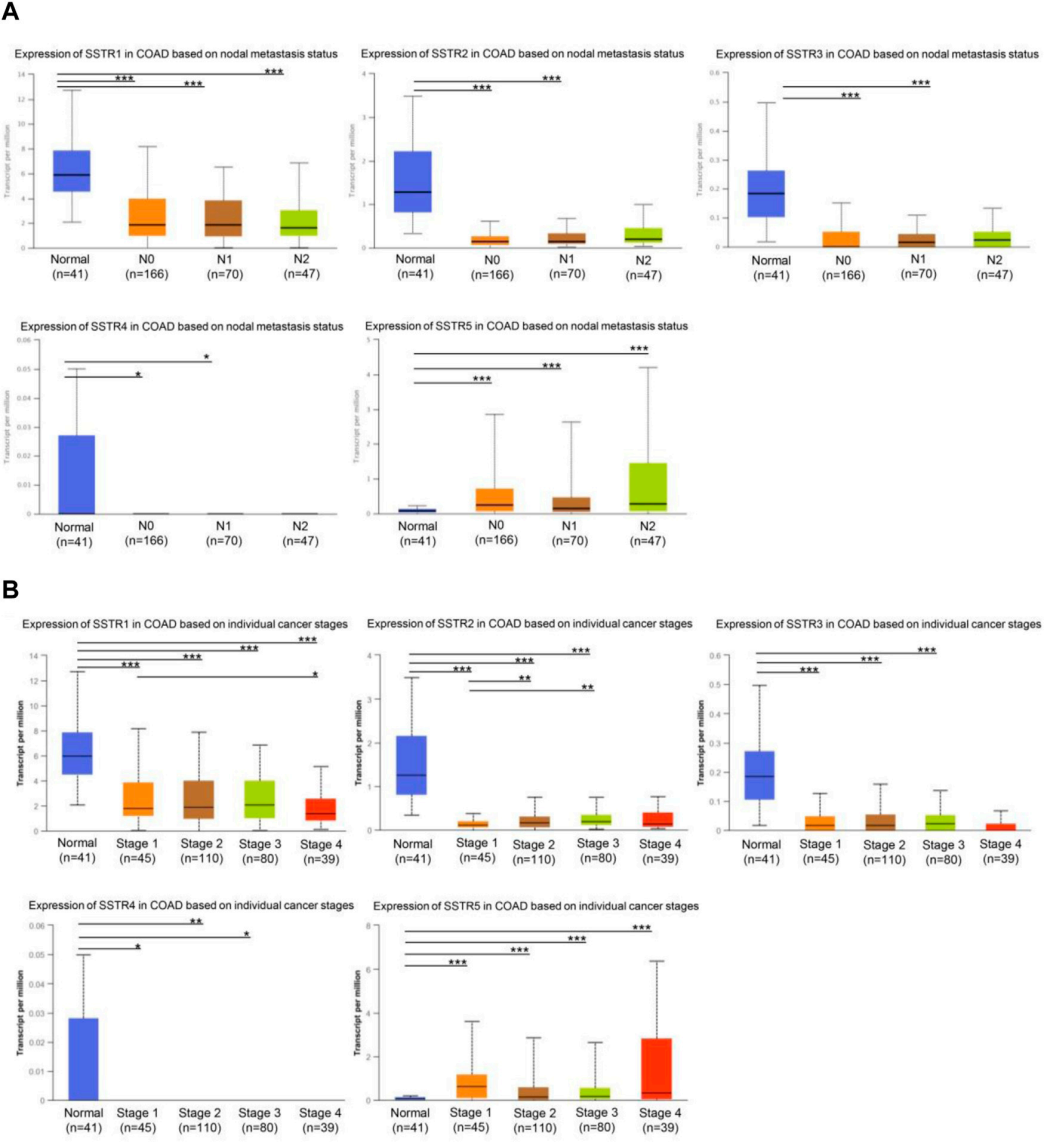
Relationship between stage and lymph node metastasis of COAD and SSTR family members. **(A)** Relationship between SSTR family mRNA expression levels and lymph node metastases of patients with COAD. Relationship between SSTR family mRNA expression levels and cancer stage of patients with COAD. **(B)** Relationship between SSTR family mRNA expression levels and cancer stage of patients with COAD. *p < 0.05, **p < 0.01, ***p < 0.001 compared with controls. COAD, Colon adenocarcinoma; SSTR, somatostatin receptor; UALCAN, University of Alabama at Birmingham Cancer Data Analysis Portal.

First, we investigated whether COAD staging and lymph node metastases were correlated with SSTR family member expression levels. In tumors with lymph node metastases at the N0-N2 stage, the mRNA expression levels of the four SSTR family genes (all but SSTR5) were lower (Figure 3A). Furthermore, compared to normal tissue, tumor stage 1–4 subgroups exhibited lower SSTR1-4 mRNA expression levels. A correlation occurred between SSTR1-2 expression and the different COAD stages (Figure 3B). These results suggested that SSTRs (SSTR2 in particular) contribute to COAD development.

Next, we examined the association between the clinicopathological characteristics of COAD and SSTR family gene expressions in The Cancer Genome Atlas Program database. Statistical analysis of the clinicopathological characteristics of 237 patients with COAD showed a correlation between SSTR1 expression and sex. SSTR4 expression was linked to age, N stage, and clinical stage, while SSTR1/2 expressions were substantially linked to lymphatic invasion (*p* < 0.05; Table 2).

**TABLE 2:** Clinicopathologic parameters of and SSTR family member expressions in COAD.

Characteristics	N	SSTR1			SSTR2			SSTR3			SSTR4			SSTR5		
		Low	High	*p*	Low	High	*p*	Low	High	*p*	Low	High	*p*	Low	High	*p*
**Gender**				**0.011**			0.885			0.997			0.660			0.434
Male	117	84	33		75	42		79	38		97	20		90	27	
Female	120	67	53		78	42		81	39		102	18		87	33	
**Age (year)**				0.532			0.887			0.471			**0.036**			0.294
≤60	72	48	24		46	26		51	21		55	17		57	15	
>60	165	103	62		107	58		109	56		144	21		120	45	
**T stage**				0.358			0.500			0.626			0.923			0.169
T1 + T2	45	26	19		31	14		29	16		38	7		30	15	
T3 + T4	192	125	67		122	70		131	61		161	31		147	45	
**N stage**				0.369			0.482			0.886			**0.032**			0.483
N0	137	84	53		91	46		93	44		121	16		100	37	
N1 + N2	100	67	33		62	38		67	33		78	22		77	23	
**M stage**				0.298			0.596			0.061			0.204			0.226
Mx + M0	208	130	78		133	75		136	72		177	31		158	50	
M1	29	21	8		20	9		24	5		22	7		19	10	
**Pathologic stage**				0.411			0.612			0.969			**0.044**			0.582
Stage I + II	135	83	52		89	46		91	44		119	16		99	36	
Stage III + IV	102	68	34		64	38		69	33		80	22		78	24	
**lymphatic invasion**				**0.006**			**0.048**			0.118			0.288			0.100
**+**	82	62	20		46	36		50	32		66	16		56	26	
**-**	155	89	66		107	48		110	45		133	22		121	34	

Bold font indicates significant difference.

### Prognostic significance of SSTR gene family in patients with COAD

**FIGURE 4:**
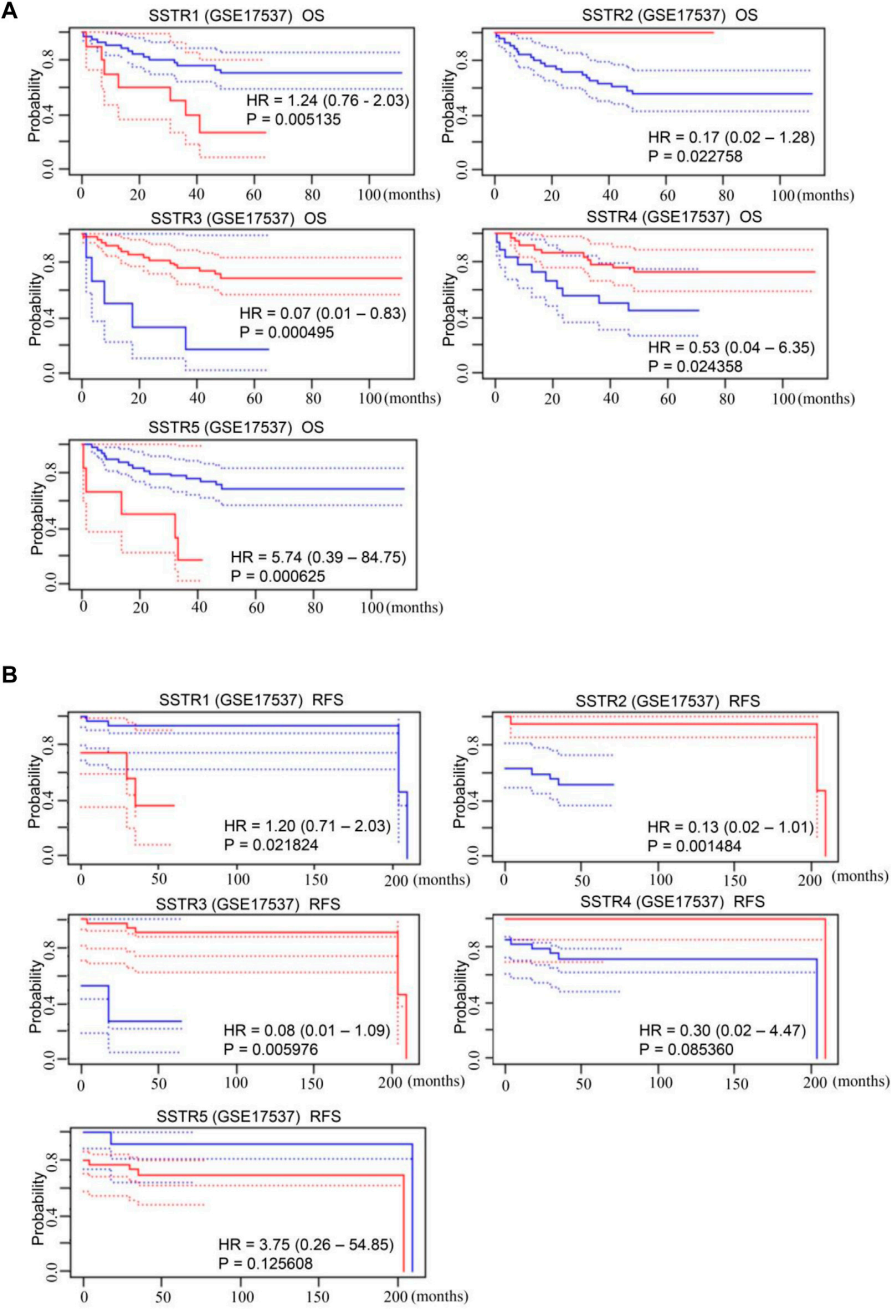
Prognostic value of mRNA expression levels of SSTR family members in patients with COAD. **(A,B)** The PrognoScan database was used to analyze the OS and DFS of the SSTR family in patients with COAD. COAD, colon adenocarcinoma; DFS, disease-free survival; OS, overall survival; SSTR, somatostatin receptor.

The SSTR family mRNA expression in patients with COAD was used to evaluate its prognostic value. A survival analysis of disease-free survival (DFS) and overall survival (OS) was performed to determine clinical prognosis. The PrognoScan database was used for the analysis. Notably, increased mRNA expressions of SSTR1 {overall survival [OS]: hazard ratio [HR] = 1.24 (0.76–2.03); *p* = 0.005} and SSTR5 [OS: HR = 5.74 (0.39–84.75); *p* = 0.001] were linked to poor OS in patients with COAD. Increased expressions of SSTR2 [OS: HR = 0.17 (0.02–1.28); *p* = 0.023], SSTR3 [OS: HR = 0.07 (0.01–0.83); *p* = 0.001], and SSTR4 [OS: HR = 0.53 (0.04–6.35); *p* = 0.024] were correlated with better OS (Figure 4A). Increased expressions of SSTR2 [DFS: HR = 1.78 (0.94–3.39); *p* = 0.005] and SSTR3 [DFS: HR = 0.08 (0.01–1.09); *p* = 0.006] were remarkably correlated with improved DFS in patients with COAD. Nevertheless, elevated SSTR1 expression [DFS: HR = 1.78 (0.94–3.39); *p* = 0.005] was substantially linked to poor DFS in patients with COAD (Figure 4B).

### Genetic alteration and functional analysis of SSTR family in patients with COAD

**FIGURE 5:**
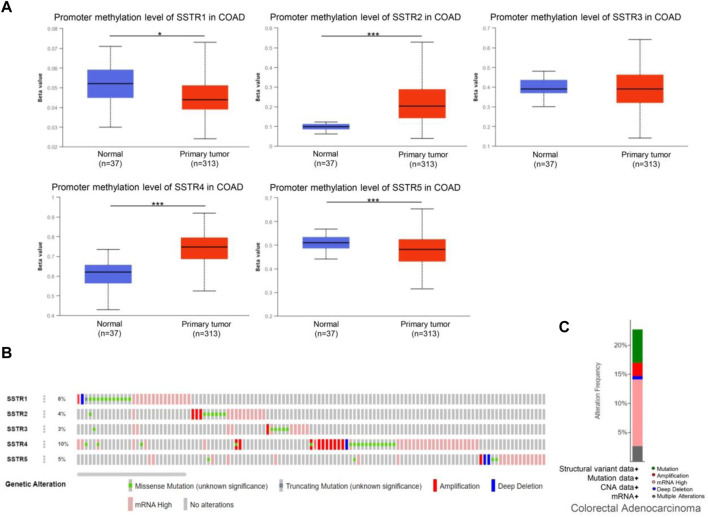
Genetic alterations and DNA methylation levels of the SSTR family members in patients with COAD. **(A)** DNA methylation changes in the SSTR family members in patients with COAD were assessed by the UALCAN database. **(B,C)** Summary of the rate of alteration for the SSTR family in the COAD (cBioPortal). *p < 0.05, **p < 0.01, ***p < 0.001 compared with control. COAD, colon adenocarcinoma; SSTR, somatostatin receptor; UALCAN, University of Alabama at Birmingham Cancer Data Analysis Portal.

DNA methylation of COAD genes is a potential epigenetic biomarker for the early detection of COAD. The UALCAN database was used to determine the methylation levels of the SSTR genes in patients with COAD. In contrast to normal tissues, COAD samples had considerably lower levels of SSTR1/5 DNA methylation but remarkably higher SSTR2/4 methylation levels. For SSTR3, there were no remarkable variations between the normal and malignant tissues (Figure 5A). We subsequently used the cBioPortal dataset to investigate genetic alterations in each SSTR family member. All five SSTR family members were altered in patients with COAD, with alteration rates of 6%, 4%, 3%, 10%, and 5%, respectively (Figure 5B). The most prevalent SSTR family abnormalities in patients with COAD were mRNA alterations and mutations (Figure 5C).

**FIGURE 6:**
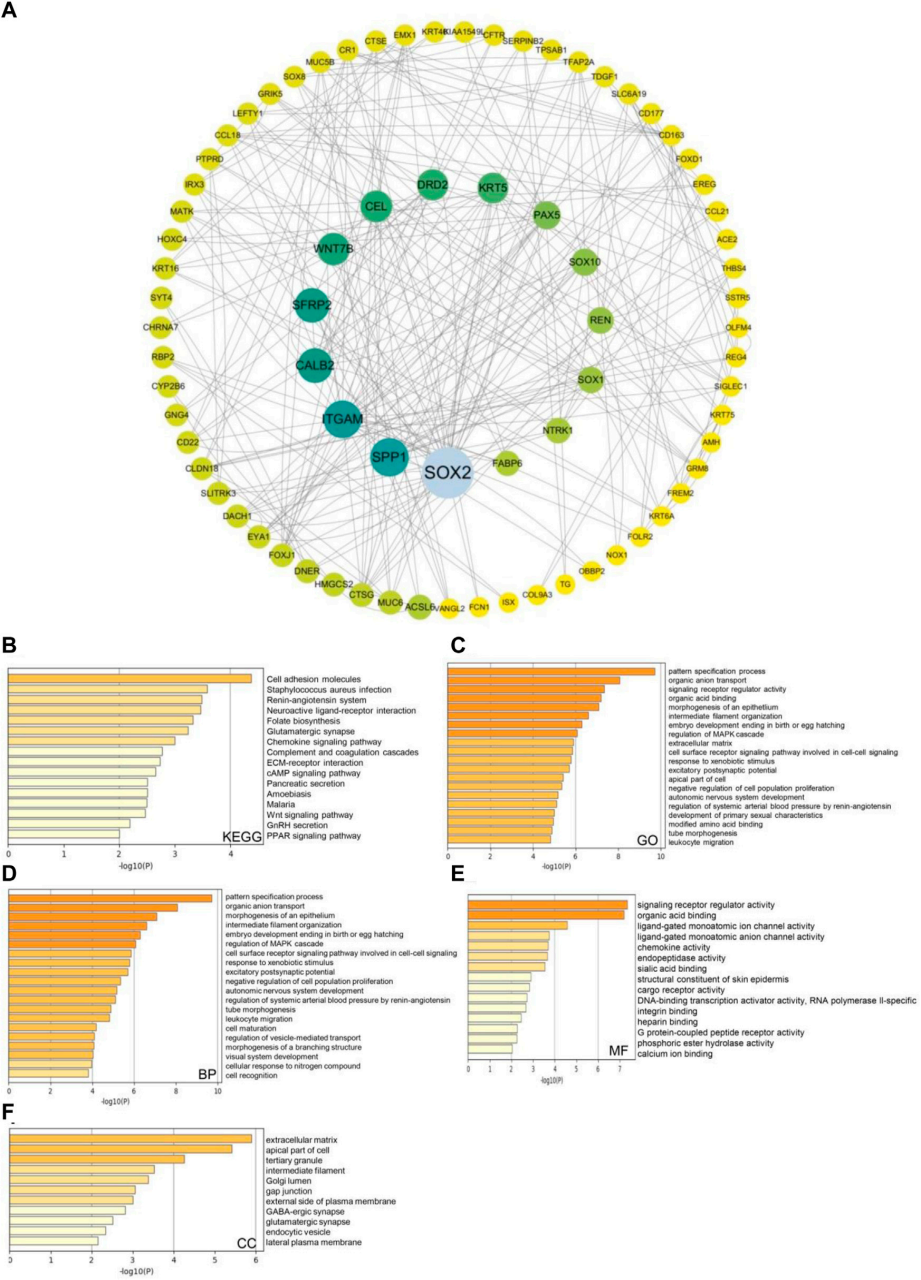
SSTRs and SSTR-associated molecules co-expressed in COAD and their predicted functions and signaling pathways. **(A)** The cBioPortal database was used to identify the 178 SSTR-associated co-expressed molecules that are most frequently altered in COAD. SSTR family members and their associated co-expressed genes were used to generate the PPI network using the Cytoscape database. **(B–F)** Functional enrichment analysis was used to analyze the biological functions of the SSTR family members and their co-expressed genes. COAD, colon adenocarcinoma; PPI, protein–protein interaction; SSTRs, somatostatin receptors.

Next, we used the Cytoscape v.3.9.0 database to identify co-expressed genes with a cutoff point of |log2 fold-change| ≥ 0.7 and *p* < 0.05. The co-expression network of key genes linked to the SSTR family was generated using the cBioPortal database (Figure 6A; Supplementary Table S1). The biological functions of the SSTR members and their co-expressed genes were assessed via GO annotation and KEGG pathway analyses using the Metascape database. For the co-expressed genes, KEGG pathway analysis was performed for cell adhesion molecules, the cAMP signaling pathway, and *Staphylococcus aureus* infection (Figure 6B). The GO findings illustrated that the co-expressed genes were primarily correlated with the pattern specification process, cell–cell signaling mediated by a cell surface receptor pathway, and signaling receptor regulatory activity (Figure 6C). These genes were primarily involved in pattern specification, epithelial morphogenesis, and MARK cascade regulation (Figure 6D). A molecular function analysis revealed the primary involvement of these genes in signaling receptor regulatory activity, DNA-binding transcription activator activity, and ligand-gated monoatomic ion channel activity (Figure 6E). According to the analysis of cellular components, these genes were often linked to the extracellular matrix and the apical region of the cell (Figure 6F).

### Association of SSTR family member expressions with immune infiltration in COAD

**FIGURE 7:**
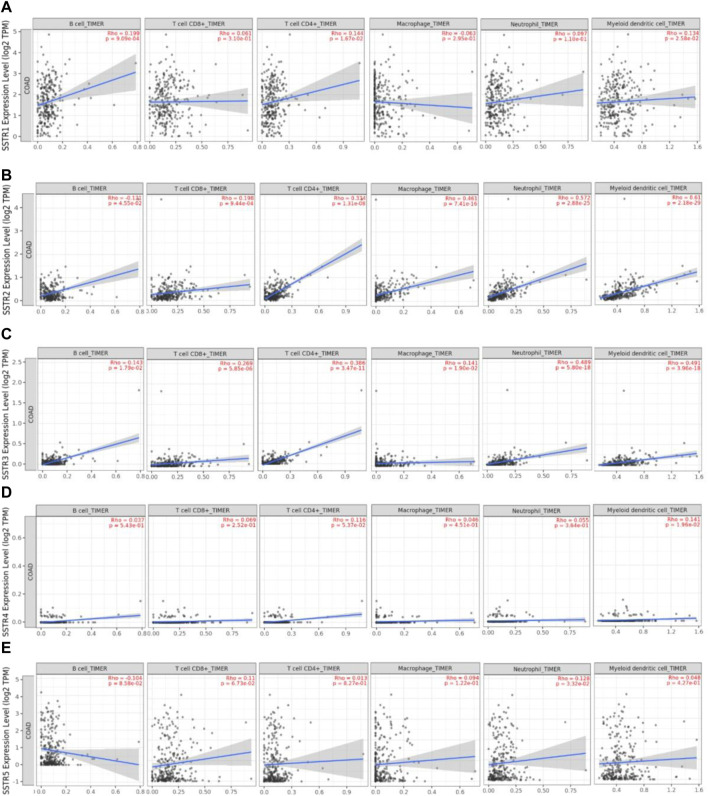
Association between SSTR mRNA expression with immune cell infiltration. **(A–E)** The TIMER2.0 database was used to evaluate the associations between SSTR family members and immune cell infiltration. SSTR, somatostatin receptor; TIMER, Tumor Immune Estimation Resource.

We used the TIMER2.0 database to investigate the correlation between the SSTR gene family and immune cell infiltration (Figure 7). SSTR1 expression was significantly associated with CD4^+^ T cell (cor = 0.102; *p* < 0.05), CD8^+^ T cell (cor = 0.169; *p* < 0.05), B cell (cor = 0.191; *p* < 0.05), DCs (cor = 0.190; *p* < 0.05), and neutrophil (cor = 0.103; *p* < 0.05) infiltration. SSTR2 mRNA expression was significantly associated with the infiltration of CD4^+^ T cells (cor = 0.370; *p* < 0.05), CD8^+^ T cell (cor = 0.169; *p* < 0.05), B cell (cor = 0.102; *p* < 0.05), macrophages (cor = 0.487; *p* < 0.05), DCs (cor = 0.475; *p* < 0.05), and neutrophils (cor = 0.499; *p* < 0.05). SSTR3 expression was significantly associated with CD4^+^ T cell (cor = 0.397; *p* < 0.05), CD8^+^ T cell (cor = 0.207; *p* < 0.05), B cell (cor = 0.233; *p* < 0.05), macrophages (cor = 0.238; *p* < 0.05), DCs (cor = 0.444; *p* < 0.05), and neutrophil (cor = 0.382; *p* < 0.05) infiltration. SSTR4 mRNA expression was significantly associated with the infiltration of CD4^+^ T cells (cor = 0.121; *p* < 0.05), macrophages (cor = 0.098; *p* < 0.05), and DCs (cor = 0.137; *p* < 0.05). No correlation was observed between SSTR5 expression and immune cell infiltration. These results suggest that SSTR family members, particularly SSTR2, potentially affect the immune response in the COAD tumor microenvironment.

We analyzed the marker types of DCs, CD8^+^ T cells, neutrophils, and tumor-associated macrophages in COAD using the TIMER2.0 database to further investigate the relationship between SSTR family expression and different immune cells (Table 3). We observed a correlation between SSTR1 and CD8^+^ T cells. We also observed a strong correlation between SSTR2 and CD8^+^ T cells, B cells, T cells, tumor-associated macrophages (TAMs), M2 macrophages, neutrophils, DCs, T helper type 1 (Th1) cells, Tfh cells, regulatory T cells (Tregs), exhausted T cells, and monocytes. A strong association occurred between CD8^+^ T cells, B cells, T cells, TAMs, M1 and M2 macrophages, neutrophils, DCs, natural killer (NK) cells, Th1, Th2, Tfh, Th17, Tregs, T-exhausted cells, and monocytes. SSTR4 expression was moderately correlated with B cells. A moderately strong correlation was observed between SSTR5 and M1 macrophage markers in patients with COAD. Furthermore, these results indicate that SSTR family members are likely to contribute to the immune infiltration of COAD.

**TABLE 3:** Correlations between SSTR family member expressions and immune cell markers.

		SSTR1		SSTR2		SSTR3		SSTR4		SSTR5	
		Cor	*p*	Cor	*p*	Cor	*p*	Cor	*p*	Cor	*p*
CD8^+^ Tcell	CD8A	0.117	*	0.343	***	0.437	***	0.022	0.662	0.068	0.174
	CD8B	0.107	*	0.230	***	0.264	***	0.106	*	0.015	0.758
	GZMA	0.101	*	0.270	***	0.346	***	0.020	0.684	0.045	0.366
B cell	CD19	0.071	0.154	0.302	***	0.477	***	0.132	**	−0.016	0.753
	CD79A	0.094	0.058	0.290	***	0.473	***	0.157	**	−0.030	0.550
	MS4A1	0.054	0.281	0.272	***	0.396	***	0.135	**	−0.047	0.346
T cell	CD3D	0.057	0.249	0.307	***	0.476	***	0.081	0.102	0.031	0.529
	CD3E	0.095	0.056	0.370	***	0.539	***	0.093	0.061	0.052	0.293
	CD2	0.062	0.214	0.348	***	0.466	***	0.052	0.293	0.016	0.746
TAM	CCL2	−0.062	0.216	0.521	***	0.260	***	0.074	0.139	−0.010	0.845
	CD68	0.124	*	0.388	***	0.350	***	0.105	*	0.158	**
	IL10	0.054	0.279	0.500	***	0.395	***	0.069	0.165	0.034	0.501
M1	IRF5	−0.019	0.698	0.310	***	0.124	*	−0.047	0.344	0.131	**
	PTGS2	0.102	*	0.243	***	0.188	***	0.002	0.968	0.136	**
	NOS2	0.205	***	−0.061	0.222	0.113	*	−0.087	0.080	0.116	*
M2	MS4A4A	0.017	0.738	0.549	***	0.368	***	0.084	0.090	0.045	0.371
	CD163	0.090	0.069	0.583	***	0.420	***	0.089	0.072	0.060	0.226
	VSIG4	0.011	0.832	0.553	***	0.321	***	0.066	0.185	0.045	0.362
Neutrophils	ITGAM	0.095	0.055	0.564	***	0.390	***	0.121	*	0.105	*
	CCR7	0.098	*	0.411	***	0.525	***	0.170	**	0.021	0.678
	SIGLEC5	0.091	0.066	0.564	***	0.392	***	0.086	0.084	0.087	0.081
DC	HLA-DQB1	−0.035	0.480	0.316	***	0.295	***	0.048	0.339	0.013	0.790
	HLA-DPB1	0.015	0.767	0.526	***	0.430	***	0.110	*	0.079	0.113
	HLA-DRA	0.011	0.830	0.461	***	0.371	***	0.043	0.383	0.014	0.782
	HLA-DPA1	0.024	0.635	0.496	***	0.416	***	0.065	0.192	0.040	0.418
	ITGAX	0.078	0.116	0.595	***	0.421	***	0.078	0.118	0.086	0.082
	CD1C	0.029	0.559	0.331	***	0.319	***	0.198	***	−0.041	0.407
	NRP1	0.143	**	0.563	***	0.306	***	0.114	*	−0.002	0.968
NK cell	KIR2DL1	−0.063	0.206	0.213	***	0.206	***	−0.027	0.585	0.050	0.315
	KIR2DL3	0.018	0.718	0.185	***	0.215	***	−0.056	0.263	0.057	0.255
	KIR2DL4	0.178	***	0.244	***	0.297	***	0.003	0.945	0.117	*
	KIR3DL1	0.028	0.579	0.235	***	0.237	***	−0.030	0.546	0.044	0.382
	KIR3DL2	0.125	*	0.221	***	0.315	***	0.039	0.432	0.045	0.361
	KIR3DL3	0.043	0.393	0.077	0.120	0.143	**	−0.034	0.495	0.023	0.649
	KIR2DS4	0.097	0.051	0.196	***	0.204	***	−0.004	0.939	−0.045	0.367
Th1	TBX21	0.094	0.059	0.400	***	0.454	***	0.030	0.542	0.062	0.214
	STAT1	0.073	0.139	0.408	***	0.345	***	−0.028	0.580	−0.010	0.834
	STAT4	0.116	*	0.405	***	0.445	***	0.026	0.603	0.059	0.233
	IFNG	0.015	0.771	0.276	***	0.310	***	−0.089	0.073	0.022	0.663
Th2	STAT6	0.096	0.053	0.066	0.181	0.254	***	0.070	0.158	0.093	0.060
	GATA3	0.062	0.214	0.393	***	0.436	***	0.131	**	0.059	0.236
	STAT5A	−0.002	0.966	0.331	***	0.324	***	0.002	0.963	0.034	0.495
Tfh	BCL6	0.118	*	0.401	***	0.269	***	0.057	0.250	0.097	0.051
	IL21	−0.021	0.670	0.257	***	0.209	***	0.009	0.854	−0.055	0.267
Th17	STAT3	0.165	**	0.321	***	0.282	***	0.021	0.673	−0.109	*
	IL17A	−0.067	0.180	−0.082	0.101	0.128	*	−0.074	0.135	−0.100	*
Treg	FOXP3	0.057	0.255	0.470	***	0.529	***	0.139	**	0.058	0.247
	STAT5B	−0.010	0.839	0.354	***	0.196	***	0.093	0.060	−0.059	0.235
	CCR8	0.045	0.371	0.433	***	0.423	***	0.127	*	−0.032	0.518
T exhaustion-cell	PDCD1	0.126	*	0.358	***	0.518	***	0.105	*	0.104	*
	CTLA4	0.110	*	0.465	***	0.516	***	0.086	0.085	0.000	0.993
	HAVCR2	0.023	0.638	0.570	***	0.372	***	0.069	0.165	0.043	0.386
	LAG3	0.112	*	0.405	***	0.475	***	0.009	0.856	0.113	*
Monocyte	CD86	0.022	0.654	0.567	***	0.375	***	0.087	0.081	0.032	0.520
	C3AR1	0.037	0.452	0.582	***	0.386	***	0.074	0.134	0.031	0.528
	CSF1R	0.103	*	0.574	***	0.475	***	0.137	**	0.065	0.188

*p < 0.05, **p < 0.01, ***p < 0.001.

### Analysis and experimental verification of the potential value of SSTR2 expression in CRC immunotherapy

**FIGURE 8:**
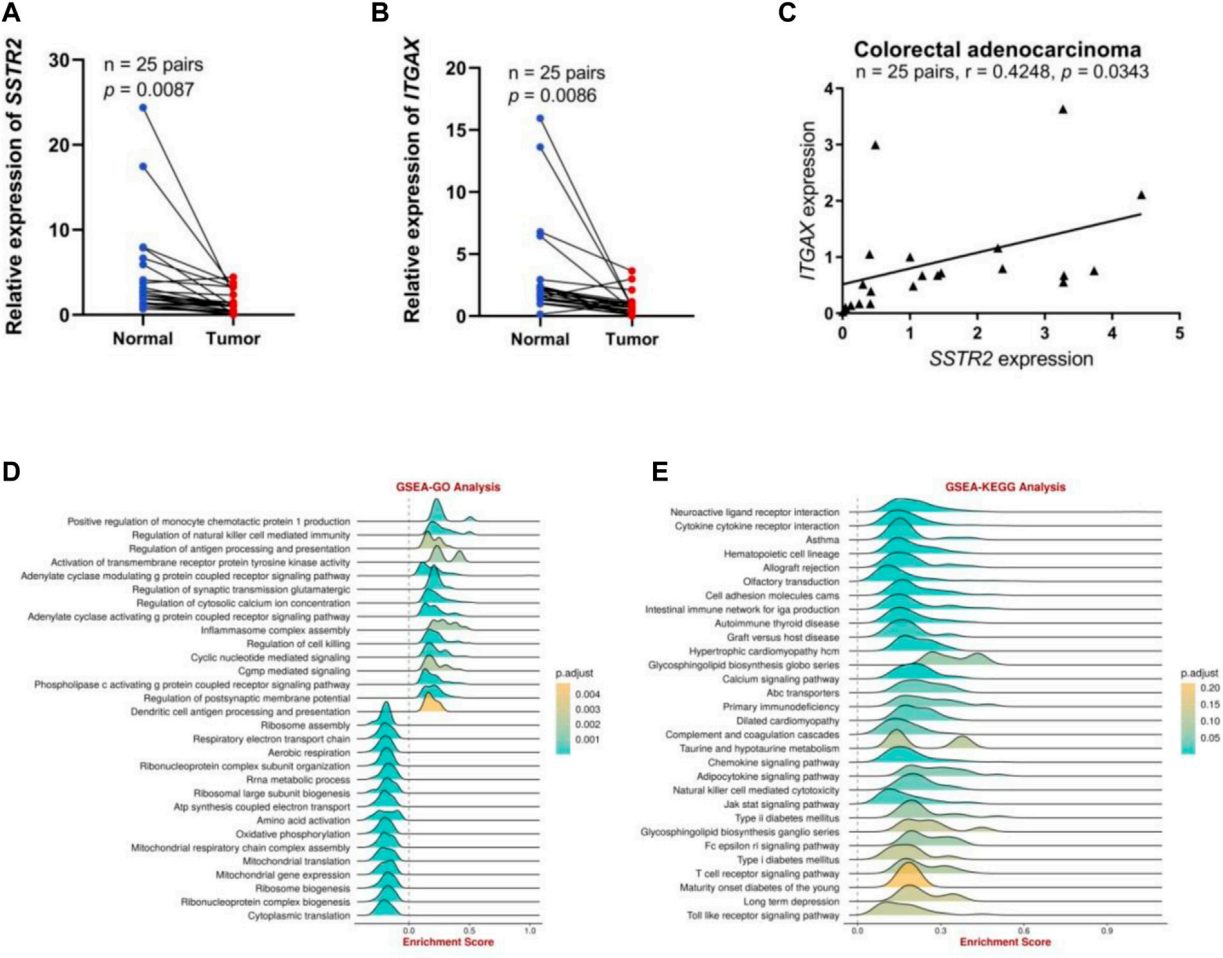
The SSTR2 and ITGAX mRNA expression levels and functional enrichment of SSTR2 in COAD. **(A)** SSTR2 mRNA expression in COAD. **(B)** ITGAX mRNA expression in COAD. **(C)** Relationships between SSTR2 and ITGAX mRNA expression levels in COAD. **(D,E)** Functional enrichment analysis of SSTR2. COAD, colon adenocarcinoma; ITGAX, integrin subunit alpha X; SSTR, somatostatin receptor.

We performed preliminary experiments to verify the differential expression of SSTR2 in COAD and its correlation with immune cell–associated molecules to further investigate the role of SSTR2 in COAD. To characterize SSTR2 expression in COAD tissues, qRT-PCR analyses revealed that the relative levels of SSTR2 expression in 25 COAD tissue samples were significantly lower than those in the matched adjacent non-tumor tissue samples (*p* = 0.0087; Figure 8A). We also demonstrated an association between SSTR and integrin subunit alpha X (ITGAX), a marker gene of DC. The qRT-PCR analysis showed that the relative expression levels of ITGAX were significantly lower in the 25 COAD samples than in the matched para-cancerous normal samples (*p* = 0.0086; Figure 8B). Finally, we found a positive correlation (r = 0.4248; *p* = 0.0343; Figure 8C) between SSTR2 and ITGAX expressions in the 25 COAD samples. Based on the Gene Set Enrichment Analysis findings, SSTR2 was primarily positively enriched using the BEST tool of the following data. These genes are responsible for the regulation of antigen processing and presentation, inflammasomal complex assembly, regulation of cytotoxicity, and antigen processing and presentation of DCs (Figure 8D); cytokine receptor interaction; an intestinal immune network for IGA production; the chemokine signaling pathway; NK cell–mediated cytotoxicity; the Jak stat signaling pathway; the T cell receptor signaling pathway; and the Toll-like receptor signaling pathway in the KEGG analysis (Figure 8E). These results are consistent with the involvement of SSTR2 in functional immune networks in COAD.

**FIGURE 9:**
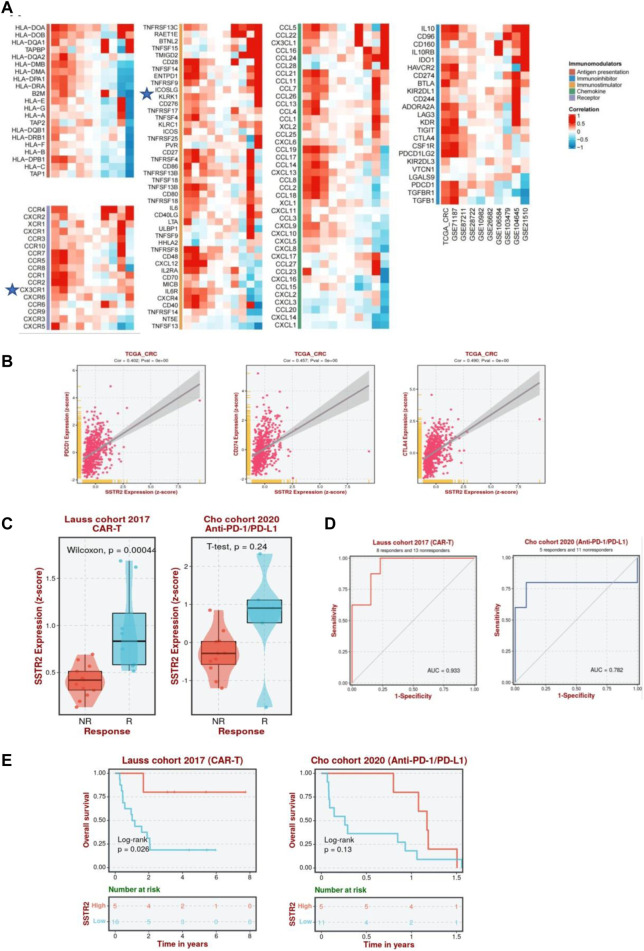
Association between SSTR2 and immunotherapy response. **(A)** Correlation between SSTR2 and immunomodulators (BEST). **(B)** Association between expressions of immune checkpoint genes PDCD1 (PD-1), CD274 (PD-L1), CTLA-4, and SSTR2 (BEST). **(C)** Expression of SSTR2 in CAR-T, anti-PD-1/PD-L1, and anti-PD-L1 responders and non-responders based on the Lauss, Cho, and IMvigor210 cohorts (BEST). **(D)** Receiver operating characteristic curve of SSTR2 for patients in Lauss, Cho, and IMvigor210 cohort (BEST). **(E)** Kaplan-Meier curves based on the Lauss, Cho, and IMvigor210 cohorts representative of the correlation between SSTR2 and OS in CRC patients receiving CAR-T, anti-PD-1/PD-L1, and anti-PD-L1 (BEST). BEST, Biomarker Exploration of Solid Tumors; CAR-T, chimeric antigen receptor T cell; CRC, colorectal cancer; CTLA-4, cytotoxic T-lymphocyte-associated antigen-4; OS, overall survival; PD-1, programmed cell death protein 1; PD-L1, programmed cell death protein ligand 1; SSTR, somatostatin receptor.

Furthermore, we analyzed the correlation between SSTR2 and immunomodulators among 10 datasets using BEST analysis, including antigen presentations, immune inhibitors, immunostimulators, chemokines, and chemokine receptors, to better understand the impact of SSTR2 on immunological responses. A substantial positive correlation between SSTR2 expression and the chemokine receptor CX3CR1; immunostimulators TNFRSFI3C and KLRKI; chemokines CCL16 and CCL1; and immune inhibitors IL10, BTLA, and KIR2DL1 are shown in Figure 9A. To further test the correlation between SSTR2 and immunotherapy, we examined whether aberrant SSTR2 expression influenced the response to immunotherapy in CRC. As shown in Figure 9B, a positive correlation was observed between the mRNA expression levels of SSTR2 and those of PDCD1 [programmed cell death protein 1 (PD-1), CD274 (programmed cell death protein ligand 1 (PD-L1)] (Huang et al., 2017), and cytotoxic T-lymphocyte-associated antigen-4 (CTLA-4) in The Cancer Genome Atlas Program dataset. SSTR2 expression was upregulated in chimeric antigen receptor T cell (CAR-T) responders in the Lauss cohort and anti-PD-1/PD-L1 responders in the Cho cohort (Figure 9C). The areas under the receiver operating characteristic curves for the Lauss and Cho cohorts were 0.933 and 0.782, respectively. This indicates that SSTR2 could discriminate between CAR-T and anti-PD-1/PD-L1 responders and non-responders (Figure 9D). High SSTR2 expression correlated with better OS in CRC patients receiving CAR-T cells in the Lauss cohort (*p* < 0.05; Figure 9E).

## Discussion

SSTRs have great potential for the diagnosis and treatment of cancer, as extensive research has illustrated the aberrant expressions of SSTR family members in several malignancies and their involvement in cancer cell proliferation and development (Rogoza et al., 2022; Aboagye et al., 2023). *In vitro* and *in vivo* studies suggest that SSTR1, SSTR2, and SSTR5 suppress the growth of pancreatic cancer (Li et al., 2005). High SSTR1 expression can silence and inhibit the proliferative rate of CRC stem cells by reducing the proliferation of acetaldehyde dehydrogenase–positive cells (Modarai et al., 2016). One preliminary study (Sun et al., 2021) confirmed that SSTR1 is a target gene affecting renal cell carcinoma (RCC) metastasis and the associated immune response and could be a prognostic biological marker of and viable therapeutic target for RCC. Another study (Zhou et al., 2009) found that the antiproliferative effects of SSTR2 were both cytostatic (growth suppression) and cytotoxic (apoptosis) by affecting the cellular apoptotic level, MAPK, and angiogenic signaling molecules in SSTR2-positive and -negative cancers. In the field of drug targeting, the delivery of nanoparticles through receptor-mediated cell interactions has received considerable attention. SSTRs are promising targets for various nanoparticles facilitated by modifying nanoparticles with specific ligands or coatings for better binding (Abdellatif et al., 2018). However, the function of SSTRs in COAD has not yet been thoroughly investigated. The five different perspectives of interest are mRNA and protein expression levels, clinical characteristics and disease prognosis, genetic mutations, pathway analysis, and immune infiltration. We extensively explained each SSTR family member’s biological impacts on COAD. Compared to nontumor cells, we discovered that SSTR1-4 mRNA and protein levels were downregulated in COAD cells. Another novel finding was that the SSTR family is strongly associated with individual clinicopathological stages and nodal involvement in COAD. These data suggest that SSTR might be correlated with COAD progression and that SSTR family members are potential diagnostic markers for COAD. Additionally, the SSTR gene family is frequently altered in COAD. Changes in mRNA expression are among the prevalent mutations. These findings strongly indicate that the differential expression of SSTR family members may be essential for COAD.

As a receptor of the G protein-coupled signal transduction pathway, SSTR can prevent proto-oncogene activation, inhibit cell proliferation, and inactivate tyrosine kinases through the MAPK pathway, thus preventing cellular proliferation (Chen et al., 2022; Sáez-Martínez et al., 2022). Next, we investigated the molecular and biological functions of SSTR family members. KEGG pathway analysis of SSTRs and their co-expressed genes revealed that cell adhesion molecules and the cAMP signaling pathway were significantly enriched. According to the GO pathway analysis, the cell surface receptor signaling pathway involved in cell–cell signaling and signaling receptor regulator activity was especially associated with SSTRs. The combination of SSTR2 or other subtypes with SST may inhibit DNA synthesis and play an antitumor role through the cAMP, MAPK, and other information pathways. This provides a fundamental principle for the development of multi-receptor SST analogs and combined therapy with signal-targeting agents such as mammalian (or mechanistic) target inhibitors of rapamycin (Robertson et al., 2022). Our results indicate that SSTR-related signaling pathways have enormous potential for antitumor immunity. Mounting data illustrate that the infiltration of immunocompetent cells may contribute to tumor development and recurrence as well as the determination of the immunotherapy response and clinical outcome (Gajewski et al., 2013; Han et al., 2022; Zou et al., 2023). SSTRs were considerably linked to six types of immune cell infiltrates: CD4^+^ T cells, CD8^+^ T cells, DCs, macrophages, neutrophils, and B cells.

SSTR2 is one of the most abundant SSTRs and a member of the GPCR family (Wu et al., 2020). Studies have shown that SSTR2 is associated with tumorigenesis in stomach and breast cancer and overexpressed in neuroendocrine tumors (Schulz et al., 2000; Skog et al., 2008; Tchoghandjian et al., 2016; Hope et al., 2018; Mohamed and Strosberg, 2019; Yin et al., 2020). SSTR2 reportedly interacts with the Wnt pathway protein Dvl1 in a ligand-independent way. SSTR2 is then targeted by Dvl1 for lysosomal degradation. SSTR2-targeted therapies may become more effective if this pathway is interfered with and SSTR2 expression in NETs is increased (Carr et al., 2023). Wildemberg showed that low SSTR2 expression can predict the failure of somatotropinomas to respond biochemically to SST analog treatment (Wildemberg et al., 2013). SSTR2 is an Epstein-Barr virus–induced druggable target in nasopharyngeal cancer, and Lechner and others demonstrated the preclinical effectiveness of targeted treatment (Lechner et al., 2021). The researchers found that the combination of SST and SSTR2 inhibits cytokine release from immune cells and impacts the tumor microenvironment (TME) (Patel, 1999; Cordova and Kurz, 2020). However, the relationship between SSTR2 and TME in COAD has not been reported. To further confirm the differential expression of SSTR2 in COAD and its association with immune cells. We collected 25 pairs of paraffin-embedded archived COAD specimens and matched adjacent normal tissues to confirm the differential expression of SSTR2 in COAD and its correlation with immune cells. In patients with COAD, SSTR2 expression is significantly downregulated. SSTR2 expression is also positively correlated with ITGAX, a gene associated with DCs. These results suggest that SSTR2 may exert antitumor effects by interacting with DCs in COAD.

Surgical treatment supplemented with postoperative adjuvant chemotherapy can treat mid-to early-stage COAD. Chemotherapy alone or combined with targeted therapy is the primary treatment for patients with advanced COAD. With only a 15% 5-year survival rate, patients with advanced COAD have a poor prognosis. Therefore, new treatments are urgently required to prolong patient survival (Auclin et al., 2017; Dekker et al., 2019). As a novel and potent anticancer therapy, immunotherapy is anticipated to become an alternative treatment option for CRC patients. The treatment of CRC has entered a new era with the advent of immunotherapy as a ground-breaking intervention following surgery in combination with radiotherapy, targeted therapy, and chemotherapy. ICI therapy is the most crucial immunotherapy in the fight against CRC (Zou et al., 2021; Pang et al., 2023).

Immunotherapies other than ICI have emerged rapidly in recent years, including CAR-T and oncolytic virus-based immunotherapy. The low immune cell infiltration level is the primary cause of the poor immune response to CRC (Hege et al., 2017; Zhao et al., 2022). The most crucial and effective therapeutic method for resolving this issue is CAR-T cell therapy. Zhang et al. (2017) performed a phase I clinical study of CAR-T therapy for CRC with high carcinoembryonic antigen expression. Of 10 patients, seven progressed while receiving prior treatment and remained stable. After CAR-T treatment, the condition of each remained unchanged. Two patients showed tumor shrinkage. In addition, Mandriani et al. showed that anti-SSTR CAR-T cells are highly effective target-dependent cytotoxic agents against a range of NET cell lines with different SSTR2/5 expression levels (Mandriani et al., 2022). This study aimed to determine whether abnormal SSTR expression affects the immunotherapy response of COAD. Our results suggest that SSTR2 may serve as an immunotherapeutic target in the treatment of COAD. The Lauss cohort showed a significant upregulation of SSTR2 expression in CRC patients receiving CAR-T cells. Differences were considered statistically significant. In patients with CRC receiving CAR-T and anti-PD-1/PD-L1 immunotherapy, SSTR2 could better discriminate between immune responders and non-responders (areas under the receiver operating characteristic curve, 0.933 and 0.782, respectively), and patients receiving CAR-T cells had a better OS, with statistically significant differences. By demonstrating a strong correlation between SSTR2 and immune molecules in CRC, our results support an immunoenhancing function of SSTR2. Additionally, our results suggest that SSTR2 contributes to tumor immunity. Therefore, it is a potential biological marker for anticipating the prognosis and effectiveness of immunotherapy in CRC patients.

Our study provides the first analysis of the relationship between SSTR family expression, tumor immune infiltration, and COAD prognosis to increase our understanding of the critical functions of these genes as drivers of tumor development and the immune system in patients with COAD. Our study identified the SSTR family as useful biomarkers and therapeutic targets that can be used to develop diagnostic and prognostic approaches to improve treatment outcomes. However, this study has some important limitations that require consideration. Furthermore, *in vitro* clinical studies are required to validate the likely processes underlying the action of multiple SSTR genes in COAD, the molecular links between them, and their clinical applications.

## Data Availability

The original contributions presented in the study are included in the article/Supplementary Material, further inquiries can be directed to the corresponding author.

## Abbreviations

COAD, Colon adenocarcinoma; CRC, Colorectal cancer; SSTR, somatostatin receptor; GPCR, G protein-coupled receptor; SST, somatostatin; KEGG, Kyoto Encyclopedia of Genes and Genomes; GO, Gene Ontology; OS, overall survival; DFS, disease-free survival; HR, hazard ratio; TIMER, Tumor Immune Estimation Resource; qRT-PCR, quantitative real-time polymeras chain reaction; DC, dendritic cell; BEST, Biomarker Exploration of Solid Tumors; AC, adenylate cyclase; CNS, central nervous system; TAMs, tumour-associated macrophages; Th1, T helper type 1 cells; Tregs, regulatory T cells; NK, natural killer cells; PDCD1, PD-1, Programmed cell death protein 1; CD274, PD-L1, Programmed cell death protein 1; CTLA-4, Cytotoxic T-lymphocyte-associated antigen-4; ICI, Immune checkpoint inhibitor; AUC, area under the receiver operating characteristic curve; UALCAN, University of Alabama at Birmingham Cancer Data Analysis Portal; PPI, protein-protein interaction.
